# Automatic segmentation software in locally advanced rectal cancer: READY (REsearch program in Auto Delineation sYstem)-RECTAL 02: prospective study

**DOI:** 10.18632/oncotarget.9938

**Published:** 2016-06-10

**Authors:** Maria A. Gambacorta, Luca Boldrini, Chiara Valentini, Nicola Dinapoli, Gian C. Mattiucci, Giuditta Chiloiro, Danilo Pasini, Stefania Manfrida, Nicola Caria, Bruce D. Minsky, Vincenzo Valentini

**Affiliations:** ^1^ Department of Radiation Oncology, Sacred Heart Catholic University of Rome, Rome, Italy; ^2^ Varian Medical Systems, Product Manager, Clinical Solutions, Palo Alto, CA, USA; ^3^ Department of Radiation Oncology, MD Anderson Cancer Center, Houston, TX, USA

**Keywords:** automatic delineation software, rectal cancer, rectal cancer delineation, independent check, clinical use of automatic delineation software

## Abstract

To validate autocontouring software (AS) in a clinical practice including a two steps delineation quality assurance (QA) procedure.

The existing delineation agreement among experts for rectal cancer and the overlap and time criteria that have to be verified to allow the use of AS were defined.

Median Dice Similarity Coefficient (MDSC), Mean slicewise Hausdorff Distances (MSHD) and Total-Time saving (TT) were analyzed.

Two expert Radiation Oncologists reviewed CT-scans of 44 patients and agreed the reference-CTV: the first 14 consecutive cases were used to populate the software Atlas and 30 were used as Test.

Each expert performed a manual (group A) and an automatic delineation (group B) of 15 Test patients.

The delineations were compared with the reference contours.

The overlap between the manual and automatic delineations with MDSC and MSHD and the TT were analyzed.

Three acceptance criteria were set: MDSC ≥ 0.75, MSHD ≤1mm and TT sparing ≥ 50%.

At least 2 criteria had to be met, one of which had to be TT saving, to validate the system.

The MDSC was 0.75, MSHD 2.00 mm and the TT saving 55.5% between group A and group B. MDSC among experts was 0.84.

Autosegmentation systems in rectal cancer partially met acceptability criteria with the present version.

## INTRODUCTION

The role of preoperative radiotherapy (RT) in rectal cancer has been well established in randomized clinical trials (RCT), and even at RCTs meta-analysis level [1]. Although the subsites of irradiation are generally agreed, the boundaries of rectal CTV still remain controversial. Moreover, volume delineation is a major source of systematic error even with advanced RT techniques [2, 3], as the magnitude of this uncertainty could be determined by several factors: imaging techniques used for delineation, different technical approaches and the use of different guidelines [4–9].

A disagreement for CTV delineation in rectal cancer up to 1 cm is reported in literature, representing one of the most significant geometric uncertainties and causes of systematic error through treatment [8, 9]. The sites of major discrepancy can be recognized in the upper anterior and inferior parts of the mesorectum [9, 10]. Many efforts are currently ongoing to reduce these discrepancies and create a common and agreed ontology among delineators: the use of international/national contouring guidelines, Quality Assurance (QA) procedures as well as radiation oncologists (RO) training showed a reduction of these geometrical uncertainties [8, 9, 11].

The recent experience reported by Joye et al [11], shows that a central platform that enhances QA in rectal cancer CTV delineation before treatment is feasible and effective. Moreover, the constant use of contouring atlas/guidelines improved the quality and homogeneity of delineations reducing this source of error.

In order to manage these uncertainties, a RT QA program for delineation has been established in our Department since 2000 [12]. The delineation process comprises two-step: a) a first operator (usually a resident or young consultant in radiation oncology) manually performs an initial segmentation for CTV and Organs at Risk (OARs); b) a second operator (expert in the specific anatomical site) revises it performing the Independent Check (IC). Although this procedure enhances quality control in the delineation of target volumes and OARs, it significantly increases the time needed for planning.

The impact of auto-segmentation systems in reducing contouring variability and increasing time sparing has recently been object of several investigations: the observed overlap, usually quantified with the Dice Similarity Coefficient (DSC), varies from 0.70 to 0.89, while time saving can be up to 50% for pelvic CTV delineation [13–17].

These results led to criticisms of this approach even if a benchmark to determine the effectiveness of these systems has not been yet established in rectal cancer and none of the studies present in literature has tested the software as part of a QA procedure.

In our previous pilot study, renamed after its publication READY (REsearch program in Auto Delineation sYstems) RECTAL-01 [15], we tested the reliability of an auto-segmentation software for CTV, OARs (bladder and femoral heads) and pelvic subsites (presacral space and mesorectum, obturator nodes, internal and external iliac nodes), in 14 patients with rectal cancer. We observed that autosegmentation is helpful in reducing the amount of time required for delineation (34%) and has acceptable overlapping values for the CTV: MDSC=0.70 and MSHD=1.13mm.

Furthermore, our previous investigation provided a first evaluation of contouring agreement among radiation oncologists from the same institution in rectal cancer: MDSC=0.75 [15] and MSHD=0.76mm (MSHD unpublished data).

Aim of the present paper, called READY RECTAL-02, is to validate the possibility to replace the first operator of the delineation workflow, analyzing similarity indices and time saving values in a larger sample of patients involving only expert radiation oncologists in the delineation phase and always maintaining a QA procedure.

## RESULTS

The geometric parameters measuring CTV overlap between automatic delineation and MC were MDSC = 0.75 (1 SD ± −0.09) and MSHD = 2.00mm (1 SD ±1.76), as shown in Table 1.

**Table 1:** Geometrical overlap and time analysis for the two groups analyzed

OVERLAPPING ANALYSIS			
	Group A (manual)	Group B (automatic)	
MDSC^¶^ ± 1SD^*^	0.84 (±0.03)	0.75 (±0.09)	
MSHD° (mm) ± 1SD^*^	0.87 (±0.56)	2.00 (±1.73)	
**TIME ANALYSIS**			**p value**
T1 (min)	13.12^ᶴ^(±4.84)	1.12^ᶷ^ (±0.44)	<0.0001 (95% CI 8,32-12,43)
T2 (min)	10.20^ᶲ^ (±5.15)	9.72^ᶲ^ (±8.67)	
TT (Total Time) (min)	23.32^†^ (±8.67)	12.84^†^ (±4.98)	

MSHD°=mean of the slicewise Hausdorff distances;

¶ MDSC= Median Dice Similarity Coefficient;

* 1 Standard Deviation;

TT=Total Time (min);

ᶴ Manual segmentation time (min);

ᶷ Autosegmentation time (min);

ᶲ Independent Check time (min).

The analysis of TT showed a 55.5% (10.38 min) time saving: TT was 12.8 min in the automatic delineation group (group B) vs 23.3 min in the manual one (group A) with p<0.0001 (see Table 1).

Analyzing the CT images, we noticed that the two test patients with the poorest MDSC and MSHD between auto-segmentation and MC presented “irregular” pelvic anatomy.

The first patient had numerous bowel loops in the pelvis and the second one presented with an 8 cm uterine fibroma.

Therefore, since 2 of the 3 criteria were met, the SmartSegmentation-Knowledge-Based-Contouring software v. 13.5 (Varian Medical Systems – Palo Alto, California, U.S.A.) (SS-KBC) could be considered acceptable for clinical practice as a first step of the locally advanced rectal cancer CTV delineation procedure in the framework of our departmental QA program.

The MDSC and MSHD among experts for the 30 test patients were 0.84 and 0.87 mm, respectively. These values can therefore represent a reliable intra-institutional expert-based benchmark in rectal cancer district.

## DISCUSSION

The aim of the study was to prospectively validate the use of autocontouring-systems in a clinical practice in which a two steps procedure for delineation QA is regularly performed (manual delineation by a first operator followed by independent check of a second one) for locally advanced rectal cancer patients.

Different steps had to be completed to test the effectiveness of the software: firstly we analyzed the reliability of the software in running a first delineation for CTV in locally advanced rectal cancer. The CTV delineation was performed as described by Valentini et al [18] and represented our target volume ontology.

Relevant clinical and anthropometric data have been inserted in the interactive database form of the library. The atlas was finally populated selecting the first 14 consecutive patients included in the study. The number of patients selected as atlas was arbitrary, since to our knowledge there were no studies defining the number of patients needed to populate an atlas library for autosegmentation purposes.

The other 30 patients were included as test patients and for all of them (44) the CTV was agreed between two expert RO.

At a later stage we defined an intra-institutional performance threshold to introduce the SS-KBC in clinical practice, based on the results of the pilot study [15], and set the geometrical and time parameters to be met:

- MDSC ≥ 0.75
- MSHD ≤ 1mm
- TT (min) sparing ≥ 50%

The strengths of this investigation, when compared to other publications in this field present in literature [13, 14, 17], can be recognized in a clear shared and agreed definition of the ontology (Supplementary Table 1) and in the definition of the anatomical parameters taken into consideration for patients' selection (Supplementary Table 2a and 2b).

The obtained positive results, show that there is benefit in the use of this software (the reported time sparing was 55.5%) with an acceptable reliability, even if an IC should always be performed.

Furthermore, the experts reached an agreement of 0.84 while the software of 0.75: the overlap difference can be therefore quantified in 0.09 (9% of MDSC): This observation gains more importance considering that up to date neither studies on automatic segmentation software [13–17], nor experiences conducted among different groups of RO [8, 9, 11], demonstrated a perfect agreement among experts.

For these reasons the “gold standard” to be achieved could not be represented by a 100% overlap (MDSC = 1), but it should be represented by the intra-institutional agreement threshold.

An analysis has also been conducted to verify if the different thickness of the CT slices (5 mm vs 2.5 mm) determined outcome differences: no disagreement was observed for the geometrical overlap, while a statistical significant difference has been recognized in the IC procedure, which was longer in the 2.5 mm slice thickness group (see Supplementary Table 3 for time values).

The system seems then to be reliable, even when the spatial resolution to propagate structures set is not the same, but longer time is of course required to revise the proposed contours.

As the observed values of MDSC and MSHD were 0.75 and 2 mm respectively. The MSHD value seems disappointing when compared to READY-RECTAL-01 results (where it was 0.76 mm): this is probably related to the fact that Hausdorff distance is based on linear distances between two planar sets and is particularly revealing even if the test contour differs substantially from the reference one only in a very small region, while area indices (such as the MDSC) are generally speaking “forgiving” of small, local deviations between the two segmentations [21].

A qualitative analysis was therefore conducted and excluded the two outlier patients. Eliminating these patients, the MDSC in group B increased to 0.77 (1 SD ± 0.09) and MSHD decreased to 1.58 mm (1 SD ± 0.7) (see Supplementary Table 4).

A potential weakness of this study is that the OARs (small bowel, bladder and femoral heads) were not taken into account. We chose not to consider them for the following reasons: 1) automatic segmentation is still not reliable for small bowel due to technical limits related to the extreme anatomical variability of this organ; 2) the READY RECTAL-01 results for bladder were not satisfactory and therefore we did not include this organ in the analysis of the results; 3) the femoral heads reached a good MDSC and MSHD in the automatic setting (0.83 and 0.53mm, respectively), but without any significant time saving (TT=2.4%).

Another potential bias of this investigation regards the atlas selection phase, as only a limited, even if not small, number of parameters can be inserted in the research template and the choice of the best fitting case to be propagated is only manual. This operation could decrease the reliability of the auto-segmented CTV due to inappropriate selection of the anthropometric parameters used for the choice of the best fitting atlas case. Moreover, it could limit the clinical significance of increasing the number of cases in the library and protract the autosegmentation time. However, these weak points are counteracted by the fact that the system performs the automatic segmentation very quickly and by the QA offered by the IC of the second operator.

The READY-RECTAL-02 study on locally advanced rectal cancer demonstrated that the automatic CTV segmentation performed with the SS-KBC software, overcame two of the three acceptability criteria set for its implementation in a clinical setting: MDSC≥0.75 (MDSC=0.75), TT savings ≥50% (TT savings=55.48%).

We could more safely accept the software also because the parameter that did not meet the threshold level, MSHD, can lose its significance in PTV expansion.

SmartSegmentation-KBC can therefore safely substitute the first operator in the frame of the IC contouring workflow adopted in our Department.

The use of automatic segmentation software could be an opportunity for RO to generate a shared and agreed ontology for therapy volumes definition. These components could at the end contribute in reducing the systematic error related to the delineation process, which still represents one of the most critical issues of modern radiation therapy.

In parallel to the morphological validation of the automatic segmentation, a second generation of studies should evaluate the dosimetric impact and reliability of these software, as recent experiences showed that even if high concordance with master contours is described by the different similarity indices, dosimetrical variability and significant target underdosage can be recognized [22–23].

## MATERIALS AND METHODS

### Study design

A total of 44 consecutive CT scans of patients with low-mid locally advanced rectal cancer were selected to validate the system.

The planning CT images were acquired from the third lumbar vertebra to below the lesser trochanters. For the first 29 enrolled patients slice thickness was of 5mm, while for the following 15 it was of 2.5mm. As routinely performed in our institution, all simulation images were acquired without intravenous contrast. To our knowledge, no study demonstrated a benefit obtained through contrast agent administration and this kind of acquisition did not hamper the anatomical definition of the images for autosegmentation purposes.

Two RO with expertise in rectal cancer agreed a manual segmentation of the CTV, following internal guidelines for delineation of locally advanced rectal tumor (Supplementary Table 1), according to clinical stage and tumor site [18]. The agreed delineation was named Master Contour (MC) and represents the benchmark for the following geometrical comparisons.

The first 14 patients (8 female and 6 male) were used to populate the library (defined as “atlas patients”), the other 30 were used to test the system (defined as “test patients”).

The system offers a set of parameters (clinical and anthropometric) that can be selected to facilitate the choice of the best fitting atlas patients for each individual test one. On the basis of the results of the READY RECTAL-01 [15], the following parameters were selected: stage, tumor localization, sex, age, weight, height, Body Mass Index (BMI) and fertility state.

Anthropometric characteristics, such as sacrum coccygeal distance (on the sagittal plane) and anterior superior iliac spin distance between upper iliac crests (on the axial plane) were also taken into account.

Patient characteristics' are reported in Supplementary Table 2a and 2b.

One month after the delineation of the MC, each expert performed the manual (group A) and automatic (group B) delineations of 15 of the 30 test patients and a cross IC was done.

The geometrical overlap between the automatic segmentations (group B) and the MC was then calculated, to verify the reliability of the automatic segmentation software.

In order to define the agreement between experts (which also represents our Intra-institutional benchmark), the geometrical overlap between the manual contours (group A) and the MC was calculated too.

Given the MDSC for manual CTV (0.75), MSHD (0.76mm) and TT saving of 34% obtained in READY RECTAL-01 [21], a MDSC ≥ 0.75, a MSHD ≤1mm and TT savings ≥50% were considered as threshold values to be overcome for the implementation of the system in clinical use.

### Overlap evaluation

The overlap was calculated analyzing two geometrical parameters [19, 24–26]:

1. Median Dice similarity coefficient (MDSC). The Dice similarity coefficient (DSC) is defined as the area of overlap between two sets of contours divided by their mean area (2│A∩B│/│A│+│B│). A DSC = 0 shows that there is no overlap between the analyzed structures, while a DSC = 1 describes a total overlap.
2. Mean of the slicewise Hausdorff distances (MSHD). It is obtained calculating the symmetric Hausdorff distance on each slice, and using its mean over all slices containing expert-contours. A MSHD=0 means that there was total overlap, whereas the bigger is MSHD, the less is the overlap between the two contours. The PTV margin expansion of target volumes can represent a potential bias, especially if an anisotropic margin is used, and this limitation can reduce the reliability of this measure.

### Time evaluation

To calculate the total time (TT) we followed the QA protocol daily used in our Department.

In group A (manual contour) the TT was the sum of the time for manual contouring and the time for the IC of delineation by the reviewer, while for group B (automatic contour) of the time for autodelineation (including the time needed to choose and propagate case from the atlas library to test case) and the time for the IC.

### Acceptability criteria

The achievement of 2 out of the above reported 3 criteria (TT saving plus one geometrical parameter) was considered sufficient for the introduction of the system into clinical practice.

### Statistical evaluation

Total time was tested in pairs using Student's t-test difference. Values at the 0.05 were considered as statistically significant. The statistical analyses were performed using MedCalc for Windows, version 9.5.0.0 (MedCalc Software, Mariakerke, Belgium). Since to introduce the SS-KBC into clinical practice the software had to reach the predetermined acceptability criteria, there was no need to conduct a statistical analysis between the manually delineated group and the autosegmented one.

## SUPPLEMENTARY TABLES


