# Supplementary material for: Automatic segmentation software in locally advanced rectal cancer: READY (REsearch program in Auto Delineation sYstem)-RECTAL 02: prospective study
Source: Oncotarget. 2016 Jun 10;7(27):42579–84. doi: 10.18632/oncotarget.9938 (PMC5173157; doi:10.18632/oncotarget.9938)
Supplement: Supplementary file 1 [file oncotarget-07-42579-s001.pdf]

Automatic segmentation software in locally advanced rectal cancer: READY (REsearch program in Auto Delineation sYstem)-RECTAL 02: prospective study

SUPPLEMENTARY TABLES

Supplementary Table S1: Subsites inclusion criteria in the rectal cancer CTV

	Presacral space	Mesorectum	Internal iliac nodes	Obturator nodes	External iliac nodes	Sphincter complex	Ischiorectal fossa
**cT3 high (above the peritoneal reflection)**	+	+	+				
**cT3 mid-low (at the peritoneal reflection)**	+	+	+	+		+ (when APR* required)	+ (when direct tumor infiltration)
**Any cT with massive positive internal iliac nodes**	+	+	+	+		+ (when APR* required)	+ (when direct tumor infiltration)
**Any cT with massive positive obturator nodes nodes**	+	+	+	+	+ **(include only the side involved by nodal spread)**	+ (when APR* required)	+ (when direct tumor infiltration)
**cT4 for anterior pelvic organ**	+	+	+	+	+	+ (when APR* required)	+ (when direct tumor infiltration)

*APR: Abdominal Perineal Resection

Supplementary Table S2a: Atlas patients' characteristics

	Sex	Age	Weight (kg)	Height (cm)	BMI*	Menopausal state	Sacrum-coccygeal distance (cm)	Most anterior distance between upper iliac crests (cm)
Patient # 1	Male	69	68	165	25.71	-	12.70	21.94
Patient # 2	Female	73	66	158	26.44	Menopause	10.35	21.34
Patient # 3	Male	70	68	170	23.53	-	11.58	24.58
Patient # 4	Female	79	54	144	26.04	Menopause	12.86	24.69
Patient # 5	Female	70	75	163	28.23	Menopause	12.20	21.08
Patient # 6	Female	67	52	151	22.81	Menopause	10.96	22.40
Patient # 7	Female	58	85	170	29.41	Menopause	13.37	23.81
Patient # 8	Male	56	78	177	24.90	-	14.02	24.43
Patient # 9	Female	50	68	165	24.90	Fertile	11.28	23.12
Patient # 10	Male	76	68	170	23.53	-	11.59	20.24
Patient # 11	Female	39	65	162	24.71	Fertile	11.16	21.39
Patient # 12	Female	84	50	150	22.22	Menopause	11.99	24.30
Patient # 13	Male	51	97	177	30.35	-	14.80	19.41
Patient # 14	Male	77	67	165	24.61	-	11.25	23.38

* Body Mass Index (BMI)

Supplementary Table S2b: Test patients' characteristics

	Sex	Age	Weight (kg)	Height (cm)	BMI*	Fertility state	Sacrum-coccygeal distance (cm)	Most anterior distance between upper iliac crests (cm)
Patient # 1	M	73	94	180	29.01	-	12.85	22.87
Patient # 2	M	73	92	169	32.20	-	14.22	23.02
Patient # 3	M	71	69	156	27.94	-	10.25	24.19
Patient # 4	F	72	53	153	22.64	Menopausal	13.62	26.42
Patient # 5	M	62	93	190	25.76	-	12.34	25.84
Patient # 6	M	71	81	175	26.45	-	11.97	23.64
Patient # 7	M	57	81	165	29.75	-	12.14	23.28
Patient # 8	M	71	68	166	24.68	-	13.55	21.51
Patient # 9	F	55	71	163	26.72	Menopausal	13.27	24.48
Patient # 10	M	63	72	164	26.77	-	12.32	23.02
Patient # 11	M	41	80	196	20.82	-	9.6	22.7
Patient # 12	F	48	56	158	22.43	Menopausal	14.9	23.93
Patient # 13	F	75	55	148	25.11	Menopausal	11.27	24.34
Patient # 14	F	76	76	158	30.44	Menopausal	12.60	23.53
Patient # 15	M	67	65	162	24.76	-	11.64	20.78
Patient # 16	M	53	70	172	23.66	-	12.28	24.38
Patient # 17	M	57	80	178	25.25	-	14.90	23.93
Patient # 18	M	65	90	173	30.70	-	12.09	24.00
Patient # 19	F	66	82	170	28.37	Menopausal	12.87	23.85
Patient # 20	F	66	74	155	30.80	Menopausal	11.63	22.61
Patient # 21	M	77	85	185	23.37	-	15.25	24.99
Patient # 22	F	50	78	166	28.00	Menopausal	9.64	19.18
Patient # 23	M	79	63	161	24.30	-	11.00	24.07
Patient # 24	M	67	78	167	27.97	-	10.45	24.10
Patient # 25	F	68	68	149	30.4	Menopausal	11.34	23.43
Patient # 26	M	75	80	175	26.12	-	12.71	23.02
Patient # 27	F	76	59	158	23.63	Menopausal	12.17	23.11
Patient # 28	F	58	60	166	21.77	Menopausal	9.82	23.40
Patient # 29	F	69	71	170	24.22	Menopausal	14.00	25.06
Patient # 30	F	63	80	150	24.76	Menopausal	11.64	20.78

* Body Mass Index (BMI)

Supplementary Table S3: Comparison of the B-Autosegmentation group in the 2 settings of patients acquired with two different slice thickness

	B-Autosegmentation		
	CT slice thickness 5 mm (16 pts)		CT slice thickness 2.5 mm (14 pts)
MDC	0.76		0.77
95%CI§	0.75-0.78		0.75-0.78
p-value		0.190	
TT(min)	9.3		17.39
95%CI§	7.47-10.88		13.37-19.75
p-value		0.0006	
MSHD	2.42		1.58
95%CI§	0.74-8.59		0.52-2.68
p-value		0.196	

§95% CI= 95% Confidence Interval; ¶MDC= Median Dice Coefficient; †TT=Total Time; *MSHD= Mean Slicewise Hausdorff Distance.

Supplementary Table S4: Overlap analysis excluding the two outlier patients

OVERLAPPING ANALYSIS EXCLUDING 2 OUTLIER PTS		
	**Group A (manual)**	**Group B (automatic)**
MDSC¶ ± 1SD*	0.85 (±0.03)	0.77 (±0.09)
MSHD° (mm) ± 1SD*	0.80 (±0.51)	1.58 (±0.7)

MSHD°=Mean of the Slicewise Hausdorff Distances; ¶MDSC= Median Dice Similarity Coefficient;*1 Standard Deviation.
